# Unraveling the role of preexisting immunity in prostate cancer patients vaccinated with a HER-2/neu hybrid peptide

**DOI:** 10.1186/s40425-016-0183-4

**Published:** 2016-11-15

**Authors:** Ioannis F. Voutsas, Eleftheria A. Anastasopoulou, Panagiotis Tzonis, Michael Papamichail, Sonia A. Perez, Constantin N. Baxevanis

**Affiliations:** Cancer Immunology and Immunotherapy Center, Saint Savas Cancer Hospital, Athens, Greece

**Keywords:** Prostate cancer, Cancer immunotherapy, HER-2 long peptide vaccine, Preexisting immunity, Epitope spreading

## Abstract

**Background:**

Cancer vaccines aim at eliciting not only an immune response against specific tumor antigens, but also at enhancing a preexisting immunity against the tumor. In this context, we recently reported on the levels of preexisting immunity in prostate cancer patients vaccinated with the HER-2 hybrid peptide (AE37), during a phase I clinical trial. The purpose of the current study was to correlate between preexisting immunity to the native HER-2 peptide, AE36, and expression of HLA-A2 and -A24 molecules with the clinical outcome. Additionally, we investigated the ability of the AE37 vaccine to induce an antitumor immune response against other tumor associated antigens, not integrated in the vaccine formulation, with respect to the clinical response.

**Methods:**

We analyzed prostate cancer patients who were vaccinated with the AE37 vaccine [Ii-Key-HER-2*/neu*_(776–790)_ hybrid peptide vaccine (AE37), which is a MHC class II long peptide vaccine encompassing MHC class I epitopes, during a phase I clinical trial. Preexisting immunity to the native HER-2*/neu*_(776–790)_ (AE36) peptide was assessed by IFNγ response or dermal reaction at the inoculation site. Antigen specificity against other tumor antigens was defined using multimer analysis. Progression free survival (PFS) was considered as the patients’ clinical outcome. Two-tailed Wilcoxon signed rank test at 95 % confidence interval was used for statistical evaluation at different time points and Kaplan–Meier curves with log-rank (Mantel-Cox) test were used for the evaluation of PFS.

**Results:**

Preexisting immunity to AE36, irrespectively of HLA expression, was correlated with longer PFS. Specific CD8^+^ T cell immunity against E75 and PSA_146–151_ (HLA-A2 restricted), as well as, PSA_153–161_ (HLA-A24 restricted) was detected at relatively high frequencies which were further enhanced during vaccinations. Specific immunity against PSA_153–161_ correlated with longer PFS in HLA-A24^+^ patients. However, HLA-A2^+^ patients with high preexisting or vaccine-induced immunity to E75, showed a trend for shorter PFS.

**Conclusions:**

Our data cast doubt on whether preexisting immunity or epitope spreading specific for HLA-class I-restricted peptides can actually predict a favorable clinical outcome. They also impose that preexisting immunity to long vaccine peptides, encompassing both HLA class II and I epitopes should be considered as an important prerequisite for the improvement of future immunotherapeutic protocols.

Protocol ID Code: Generex-06-07

National Organization for Medicines (EOF) ID Code: IS-107-01-06

NEC Study Code: EED107/1/06

EudraCT Number: 2006-003299-37

Date of registration: 07/06/2006

Date of enrolment of the first participant to the trial: Nov 1st, 2007

**Electronic supplementary material:**

The online version of this article (doi:10.1186/s40425-016-0183-4) contains supplementary material, which is available to authorized users.

## Background

Cancer immunotherapy aims to immune recognition and elimination of tumor cells and is a novel approach for effective management of patients with prostate cancer [1]. Immune tolerance and tumor-induced immunosuppression often limit beneficial immunotherapeutic protocols [2]. Therefore, active immunization with antigenic peptides is a proposed intervention that may help to overcome these obstacles. HER-2/neu (HER-2) is expressed in a high proportion of prostate tumors and overexpressed in castration-resistant patients [3–5]. HER-2_776–790_ (referred to as AE36) has been shown to induce potent immunologic responses and chemical hybridization to a tetra-peptide from the invariant chain of MHC class II molecules (Ii-key/HER- 2_776–790_ hybrid peptide or AE37) potentiates furthermore its action [6–8]. In our previous prostate cancer phase I vaccination clinical trial, we showed that AE37 not only induced potent immunologic responses [9], but also generated specific antitumor responses that could be detected even 3 years post booster inoculation [10]. Patients expressing HLA-DRB1*11 and/or HLA-A24 alleles showed an increased vaccine induced immunological response which was followed by an increased overall survival (OS) [11]. Additionally, we found that the AE37 vaccine has the potential to induce HER-2 specific CD4^+^ T helper cells (Th) that belong to the in vivo immunological memory repertoire and could even be detected 5 years after the first vaccine inoculation [12]. Retrospective analyses of our results revealed that preexisting IFNγ immunity to the vaccine correlated with OS [13].

Clinical responses in cancer immunotherapies have been reported to be associated with several post vaccination biomarkers including frequencies of antigen-specific CD8^+^ cytotoxic T lymphocytes (CTL), delayed type hypersensitivity test (DTH) and autoimmunity [14–17]. However, pre-vaccination predictive biomarkers have not been developed sufficiently until now. Preexisting host immunity to vaccine candidate peptides before vaccination, has been proposed as a predictive biomarker of this kind, as it could act as a basis for the selection of suitable peptides for vaccination, in order to induce potent anti-tumor response that could provide cancer patients with clinical benefit [14]. Sasada et al. reported a new immunotherapeutic approach named personalized peptide vaccine, in which they selected antigen (HLA)-matched vaccine peptides based on the preexisting host immunity before vaccination and conducted phase I and II clinical trials, with improved antigen-specific immune responses and promising clinical outcomes [15].

CD4^+^ Th cells have been shown to play a pivotal role in antitumor immunity [18–21]. They help priming of tumor specific naïve CD8^+^ T cells, maintenance of CD8^+^ T cell memory and prevention of tolerance. They also induce proliferation and differentiation of CD8^+^ T cells into tumor specific effector CTLs capable for infiltration into tumor microenvironment [22, 23]. Vaccination with a HER-2 helper peptide could elicit tumor specific CTLs via cross-presentation [24]. Hence, vaccine induced immune responses might be directed not only against the targeted epitope but also against a broad range of tumor associated epitopes through epitope spreading [9, 25]. The AE37 peptide vaccine has also been reported to stimulate CD4^+^ Th cells rendering them capable of inducing immunologic memory and persistent stimulation of CTLs. Vaccine-induced T cells, secreting mainly Th1 cytokines, may activate dendritic cells (DCs) hosted in tumor microenvironment. Under these conditions the phenomenon of cross-presentation could be enhanced resulting in an epitope spreading [26].

In the current study we investigated a possible correlation between preexisting immunity to AE36 combined with the expression of certain HLA- molecules and progression free survival (PFS), in prostate cancer patients, who had been vaccinated with the AE37 hybrid peptide during our phase I clinical trial [9]. Additionally, we tried to answer the question of how preexisting immunity to other known tumor associated CTL antigens might affect patients’ clinical outcome and if it can be further enhanced by AE37 vaccination through epitope spreading.

## Methods

### Patient samples

The present study involves prostate cancer patients enrolled in the phase I clinical trial of the AE37 vaccine (Ii-Key/HER-2*/neu*_(776–790)_:Ac-LRMKGVGSPYVSRLLGICL-NH2), EudraCT2006-003299-37 [9, 11, 27]. The current study was approved by the Institutional Review Board of Saint Savas Cancer Hospital. Upon inform consent, patients received 6 monthly inoculations (primary vaccination series; PVS) with AE37 plus GM-CSF as immunoadjuvant, followed by a single booster inoculation 6 months after completion of PVS. As previously described, 50 ml of whole blood was obtained by venipuncture at prevaccination and at different timepoints during and after vaccinations [9]. PBMCs were isolated from blood samples by Ficoll (Biochrom) gradient separation at RT, washed twice in PBS and counted at Neubauer chamber. Viability was always >95 %. Cells were frozen in FCS/10 % DMSO at ≥1 × 10^7^ cell/vial at −20 °C for 1 h, transferred at −80 °C overnight and afterwards stored in liquid nitrogen until use. PBMCs were thawed in pre-warmed RPM1 1640 culture medium (Gibco® by Life Technologies, Europe) supplemented with 20 % FCS, 0,5 mM L-Glutamine (Gibco® by Life Technologies, Europe), and 1X antibiotic-antimycotic (Gibco® by Life Technologies, Europe, ref:15240–062)] and counted at Neubauer chamber using trypan blue. The average viability was >70 %.

HLA typing was performed by PCR-SSP (polymerase chain reaction-sequence specific primers) as described previously [11]. Patients' clinicopathological characteristics are presented in Table 1. Clinical progression was evaluated after enrolment of patients in the clinical trial. Based on the initial clinical status of each patient, clinical progression was defined as castration-resistance, metastasis (bone metastasis verified by radiographic evaluation) or death (Table 1).

**Table 1:** Clinical characteristics of patients

	Age at diagnosis	TNM	Stage	Gleason score (biopsy)	Gleason score (surgery)	PSA (pre-treatment)	Clinical status at 1st vac	HLA typing	HER-2 status	Progression from 1st vac
PR02	61	G2T3bN0M0	III	6 (3 + 3)	6 (3 + 3)	12.6	CS	A*24 A*32 DRB*07 DRB^a^15	1+	NP
PR04	64	G2T2bN0M0	II	5 (3 + 2)	5 (3 + 2)	----	CS	A*02 DRB1*11 DRB1*16	1+	NP
PR05	58	G4T3bN x M1b	IV	7 (3 + 4)	7 (3 + 4)	65	CR/M	A*24 A*29 DRB1*11 DRB1*14	1+	D (7)
PR06	61	G3T4N1M1b	IV	7 (4 + 3)	----	92	CS/M	A*02 A*02 DRB1*08 DRB1*11	1+	CR (50)
PR08	51	G2T4N1M0	IV	7 (4 + 3)	7 (4 + 3)	13.30	CS/NM	A*24 A*68 DRB1*11 DRB1^a^14	1+	CR (54)/NM
PR09	73	G4T4N + M0	IV	4 (3 + 1)	9 (5 + 4)	12.3	M	A*02 A*26 DRB1*16 DRB1*16	1+	D (18)
PR10	52	G2T3bN1M0	III	6 (3 + 3)	7 (4 + 3)	10.00	CS/NM	A*01 A*30 DRB1*04 DRB1*11	1+	CR/M (39)
PR11	73	G3T3bN0M0	III	7 (4 + 3)	7 (4 + 3)	18.85	CS/NM	A*02 A*32 DRB1*04 DRB1*07	2+	NP
PR12	55	G2T3bN x M1b	IV	7 (4 + 3)	----	>200	CR/M	A*26 DRB1*11	1+	D (40)
PR13	78	G4T3bN x M1b	IV	10 (5 + 5)	----	52.03	CS/M	A*02 A*32 DRB1*04	3+	D (9)
PR14	48	G3T3aN0M0	II	6 (3 + 3)	6 (3 + 3)	8.50	CS/NM	A*24 A*33 DRB1*03 DRB1*11	1+	NP
PR15	72	G1T1bN0M0	II	3 (2 + 1)	----	18.90	CS/NM	A*24 A*29 DRB1*11	2+	NP
PR16	64	T2bN0M0	II	4 (2 + 2)	----	13.40	CS/NM	A*24 A*32 DRB1*11 DRB1*15	1+	NP
PR17	49	G4T3bN1M1b	IV	9 (4 + 5)	----	12.00	CR/M	A*01 DRB1*11 DRB1*13	1+	D (20)
PR18	57	G4T3aN x M1b	IV	9 (4 + 5)	----	1000	CR/M	A*26 A*32 DRB1*01 DRB1*13	1+	D (21)
PR19	44	G2T3bN1M1b	IV	6 (3 + 3)	----	130.00	CR/M	A*02 A*24 DRB1*01 DRB1*09	2+	D (13)
PR20	70	G3T2aN0M0	II	8 (4 + 4)	7 (3 + 4)	7.11	CS/NM	A*02 A*03 DRB1*04 DRB1*12	2+	NP
PR21	56	G2T2aN0M0	III	8 (4 + 4)	8 (4 + 4)	9.50	CS/NM	A*02 A*03 DRB1*15 DRB1*16	1+	NP
PR22	63	G2T3bN0M0	III	6 (3 + 3)	8 (3 + 5)	6.80	CS/NM	A*02 A*30 DRB1*15	1+	NP
PR23	63	G3T2bN0M0	II	7 (3 + 4)	----	97.00	CS/NM	A*02 A*11 DRB1*04 DRB1*16	3+	LFW (12)
PR24	81	G2T2aN0M0	III	6 (3 + 3)	----	10.00	CS/NM	A*01 A*03 DRB1*11 DRB1*16	2+	NP
PR25	75	G3T2aN0M0	II	8 (3 + 5)	----	7.23	CR/NM	A*24 A*32 DRB1*04 DRB1*11	3+	NP
PR26	61	G4T3aN x M1b	IV	8 (3 + 5)	----	419.83	CS/M	A*03 A*11 DRB1*01 DRB1*16	2+	CR/M(45)
PR27	63	G4T2bN0M0	III	7 (4 + 3)	8 (4 + 4)	6.80	CS/NM	A*02 A*03 DRB1*11 DRB1*15	3+	NP
PR28	62	G3T3bN0M0	III	8 (3 + 5)	8 (4 + 4)	22.98	CS/NM	A*02 A*24 DRB1*16	3+	NP
PR29	67	G2T3bN0M0	III	6 (3 + 3)	10 (5 + 5)	7.50	CS/NM	A*03 A*24 DRB1*11 DRB1*13	1+	NP
PR30	66	G2T2bN0M0	III	6 (3 + 3)	6 (3 + 3)	6.00	CS/NM	A*24 DRB1*03 DRB1*11	2+	NP
PR31	52	G3T3bN0M0	III	8 (4 + 4)	8 (5 + 3)	6.80	CS/NM	A*02 A*24 DRB1*01 DRB1*11	2+	NP
PR32	65	G3T3bN x M1b	IV	9 (5 + 4)	----	320.00	CR/M	A*24 A*32 DRB1*07 DRB1*10	1+	NP

*NP* no progression, *M* metastatic, *NM* non metastatic, *CS* castrate sensitive, *CR* castrate resistant, *D* death, *LFW* lost in follow-up

*Clinical status by May 2014. Numbers in parentheses represent months from 1st vaccine to change in clinical status

### IFNγ ELISPOT assay

Preexisting immunity for native HER-2/neu peptide AE36 (aa776–790: GVGSPYVSRLLGICL) was evaluated in the IFNγ-based ELISPOT assay, a widely applied technique for detection of functional effector T cells [13, 17, 28, 29], as previously described [9]. Briefly, freshly isolated PBMCs were cultured in X-VIVO 15 medium (BioWhittaker, Cambrex) supplemented with 2 % AB human serum (Sigma), with the AE36 peptide (10 μg/mL) in precoated IFN-γ ELISPOT plates (MABTECH AB), in quadruplicates at 2.5 × 10^5^ cells per well. The plates were incubated at 37 °C in a humidified 5 % CO_2_ incubator for 40 h and developed as described by the manufacturer. Spots were enumerated using an ELISPOT analyzer (A.EL.VIS GmbH) and data are presented as specific spots (experimental spots minus background spots; i.e., PBMC in medium alone) per 10^6^ PBMCs. Individual values for IFNγ production were reported in our previous work [9].

### Dermal reaction

Local dermal reactions (LR) were determined 48 h after each vaccination cycle by measurement of induration, as previously described [9]. Each inoculation consisted of 500 μg of the AE37 vaccine mixed with GM-CSF as immunoadjuvant. Given the amount of the peptide along with the presence of the immunoadjuvant, we considered LR as a more appropriate method to evaluate HER2 preexisting immunity, compared to the standard DTH reaction (100 μg AE36 without GM-CSF). Beside this, it has been previously described that local reaction to the vaccination site can be used to evaluate immune responses in the context of immunomonitoring [30, 31]. Therefore, in the present study LR data represent measurements of induration presented as the orthogonal mean (mm) of the two sites of vaccine inoculation.

### Assessment of antigen-specific CD8^+^ T cells

Antigen-specific T cells were detected by fluorescent MHC-peptide dextramers, which are multimers based on a dextran backbone bearing multiple fluorescent moieties [32]. Such multimers are ideal for analyzing extremely low frequencies of antigen-specific T cells in peripheral blood in combination with multiparameter flow cytometry [33]. Frequencies of CD8^+^ cells specific for tumor associated CTL epitopes, characterized as immunogenic by others and our group [34–42], were assessed with specific MHC-peptide dextramers using flow cytometry. The aforementioned assessment of antigen specific T cells was performed on cryopreserved samples as part of a retrospective analysis.

Only the most common alleles among our patient cohort, i.e. HLA-A2^+^ (*n* = 12) and HLA-A24^+^ patients (*n* = 12, with two of them co-expressing HLA-A2) were analyzed in this study, as all other haplotypes were expressed in very few patients, impossible to give any substantial information.

PBMCs were stained with commercially available MHC dextramers: A*0201-HER2_369–377_ (KIFGSLAFL) (referred to as E75)–FITC, A*0201-PSA_146–154_ (KLQCVDLHV)-FITC, A*0201-HER2_85–94_ (LIAHNQVRQV)–PE, A*0201-HER2_435–443_ (ILHDGAYSL)-PE, A*0201-PSMA_27–35_ (VLAGGFFLL)-PE, A*0201-hTERT_540–548_ (ILAKFLHWL)-FITC, A*0201-Survivin_96–104_ (LTLGEFLKL)-PE, A*02402-PSA_153–161_ (CYASGWGSI)-FITC, A*02402-Survivin_20–28_ (STFKNWPFL)-PE (Immudex, Denmark). Briefly, PBMCs (1–3 × 10^6^) were incubated with 10 μl of MHC dextramer for 10 min in the dark at room temperature, followed by staining with specific monoclonal antibodies anti-CD3-APC (UCHT1), and anti-CD8-PerCP (SK1)(BD Biosciences, Europe) for 20 min in dark at 2–8 °C. Fluorescent minus one (FMO) samples (without the dextramer) were used as negative controls. Samples were analyzed by flow cytometry on a FACSCalibur (BD Biosciences). 50000 CD8^+^ T cells were collected, thus determining the detection limit of accurate dextramer^+^ measurement at ≥0.1 %. Data analysis was performed using FlowJo software.

### Statistical analysis

GraphPad Prism version five software was used for the statistical analysis of data. Two-tailed Wilcoxon signed rank test at 95 % confidence interval was used for statistical evaluation of patients at different time points. Kaplan–Meier curves and log-rank (Mantel-Cox) test were used for the evaluation of progression-free survival (PFS). Cut off limits for preexisting (R0) AE36 peptide-specific T cell frequencies (IFNγ producing cells in response to stimulation with AE36 at baseline) and LR at first vaccine (LR1) were calculated by cut off finder, http://molpath.charite.de/cutoff/index.jsp. In cases where the number of samples to be analyzed was <20 and thus the cut off finder could not be applied, X-tile software was used for defining the cut off for low- and high frequencies of tumor antigen-specific (dextramer-positive) CD8^+^ T cell populations [43]. Rmax represents the time point during vaccinations with the highest frequency of antigen specific T cells detected. The ratio Rmax/R0 was considered high when value was >2, i.e. when Rmax was at least twofold above R0 frequency detected. Statistically significant differences were considered when the *P* value was ≤ 0.05.

## Results

### Preexisting immunity to AE36 affects PFS

**Fig. 1:**
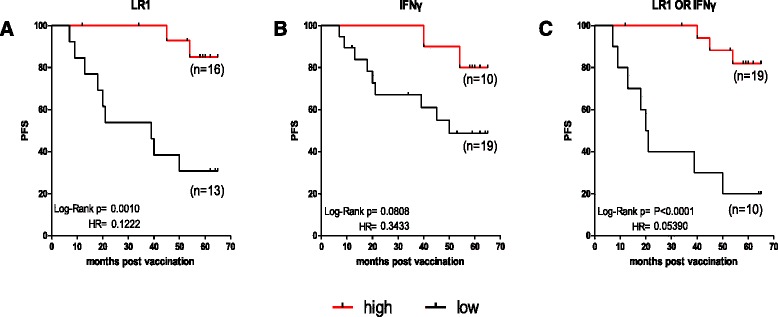
Preexisting immunity to AE36 affects PFS. Patients having high LR1 showed statistically significant longer mPFS (**a**). Patients having high IFNγ production showed a strong trend for longer mPFS (**b**). Patients having high preexisting immunity (high LR1 or high IFNγ production) showed statistically significant longer mPFS when compared to the group of patients with low preexisting immunity (**c**)

Preexisting immunity to AE36 was evaluated by two different approaches. In the first, we evaluated prior to vaccination (i.e., time-point R0) ELISPOT-based IFNγ production, whereas the second was based on the size of local (dermal) reaction (LR1) 48 h after the first vaccination. We considered LR1 as a result of preexisting immunity to AE36 simply because the time-frame of 48 h post first vaccination is not sufficient for AE37 (or any other vaccine) to induce a primary immunologic response (in this case an AE36-specific response). Using cut off finder software, the study population was divided into two subgroups, patients with high or low AE36 preexisting immunity, according to the levels of IFNγ production (high levels: ≥ 25 spots/10^6^ cells) and the size of LR1 (high: ≥10 mm induration diameter). The PFS analysis for a median follow-up time period of 58 months (range 7–65 months) estimated from the time of first vaccination is shown in Fig. 1. Median estimated PFS (mPFS) was not reached in the group of patients having high LR1 (*n* = 16), whereas mPFS in the low LR1group (*n* = 13) was 39 months [*p* = 0.001, hazard ratio (HR) =0.1222] (Fig. 1a). mPFS for patients having high IFNγ production (*n* = 10) was also not reached, whereas that for low IFNγ producers (*n* = 19) was 50 months (*p* = 0.0808, HR = 0.3433) (Fig. 1b). This significant difference for higher mPFS in the groups of patients with high LR1 or IFNγ preexisting immunity was much more intense when these patients were grouped together. Thus, mPFS in the group of patients having high LR1 and/or IFNγ preexisting immunity (*n* = 19) was, as expected, not reached being highly statistically significant (*p* < 0.0001) when compared to the group of patients with low LR1 and low IFNγ preexisting immunity (*n* = 10) (mPFS: 20.5 months; Fig. 1c).

**Fig. 2:**
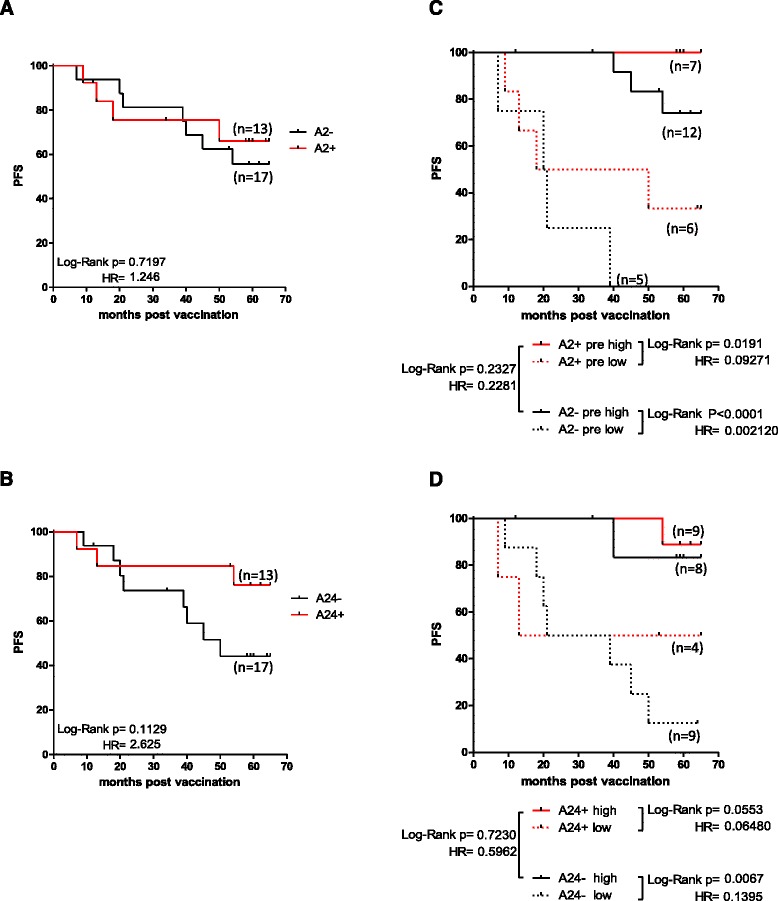
Correlation between HLA-A2 or A24 expression and preexisting immunity to AE36 with PFS. HLA-A2^+^ patients had no significant difference in mPFS compared to HLA-A2^−^ patients (**a**). However, HLA-A24^+^ patients showed a trend for increased mPFS compared to HLA-A24^−^ patients (**b**). HLA-A2^+^ patients with high preexisting immunity had statistically significant longer mPFS than HLA-A2^+^ patients with low preexisting immunity. HLA-A2^−^ patients with high preexisting immunity showed a strong trend for longer mPFS than HLA-A2^−^ patients with low preexisting immunity (**c**). HLA-A24^+^ patients with high preexisting immunity had a strong trend for longer mPFS compared to HLA-A24^+^ patients with low preexisting immunity. HLA-A24^−^ patients with high preexisting immunity showed statistically significant longer mPFS compared to HLA-A24^−^ patients with low preexisting immunity (**d**)

HLA typing was performed on the 29 patients who were included in our studies. A substantial percentage (23 out of 29) of those expressed HLA-A2 (*n* = 13) or HLA-A24 (*n* = 13), with 3 of them expressing both alleles. For the patients carrying the HLA-A2 allele, no significant difference in mPFS was observed compared to non-carriers (Fig. 2a). However, we found a trend for increased mPFS among patients expressing the HLA-A24 allele vs those who were HLA-A24^−^ (Fig. 2b). We next tried to correlate expression of HLA-A2 and HLA-A24 alleles in combination with preexisting immunity to the vaccine and mPFS. HLA-A2^+^ patients with high preexisting immunity had statistically significant longer mPFS than allele carriers with low (*p* = 0.0191), whereas a weak trend for improved PFS was observed between the HLA-A2^+^ and HLA-A2^−^ patients with high preexisting immunity. In addition, in HLA-A2^−^ patients, those with high levels of preexisting immunity exhibited statistically significant longer mPFS than those with low ones (Fig. 2c). In an analogous fashion, HLA-A24^+^ patients with high preexisting immunity had a strong trend for longer mPFS compared to those with low levels (Fig. 2d). Interestingly enough, similar mPFS was observed in patients with high preexisting immunity regardless of HLA-A24 expression (*p* = 0.7230). Finally, in HLA-A24^−^ patients, those with high preexisting immunity showed statistically significant better mPFS than those with low preexisting immunity (Fig. 2d).

To conclude, these data show that preexisting immunity to the vaccine correlates with longer PFS to all patients regardless of their HLA allele expression.

### Preexisting and AE37-induced immunity to HLA-A2 and HLA-A24-restricted CTL epitopes

Frequencies of CD8^+^ T cells specific for known immunogenic tumor associated CTL epitopes, not included in the vaccine, in patients’ peripheral blood were determined by multiparameter flow cytometry in combination with MHC-peptide dextramers at each time point (R0, R3, R6, LT). We used the following MHC-peptide dextramers: A*0201-HER2_85–94_ (HER2_85_), A*0201-HER2_435–443_ (HER2_435_), A*0201-HER2_369–377_ (E75), A*0201-PSA_146–154_ (PSA_146_), A*0201-hTERT_540–548_ (TERT), A*0201-PSMA_27–35_ (PSMA), A*0201-Survivin M2_96–104_ (SURV_96_), A*02402-PSA_153–161_ (PSA_153_), A*02402-Survivin_20–28_ (SURV_20_).

**Fig. 3:**
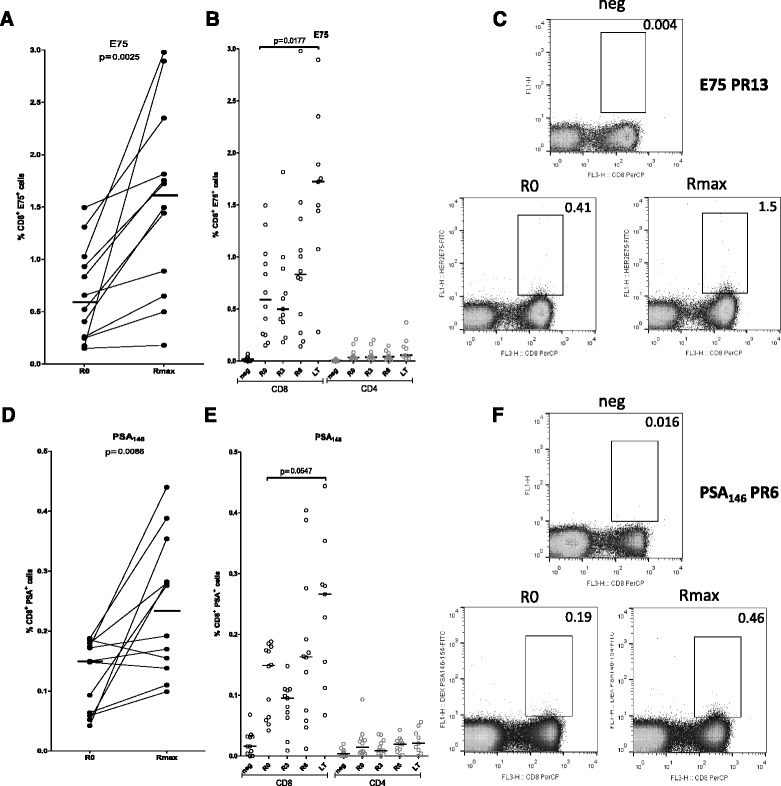
Preexisting and AE37-induced immunity to E75 and PSA_146_ in HLA-A2^+^ patients. Vaccinations induced CD8^+^/E75^+^ cells in HLA-A2^+^ patients (**a**). Frequencies of CD8^+^/E75^+^ cells at each time point (R0, R3, R6 and LT) (**b**). Representative dot plots for negative control, R0 and Rmax for patient PR13 (**c**). Vaccinations induced CD8^+^/PSA_146_^+^ cells in HLA-A2^+^ patients (**d**). Frequencies of CD8^+^/PSA_146_^+^ cells at each time point (R0, R3, R6 and LT) (**e**). Representative dot plots for negative control, R0 and Rmax for patient PR6 (**f**)

PBMCs were gated according to their FSC/SSC properties and then subgated to CD3^+^ cells which were further subgated to CD8^−^ (i.e. CD4^+^) and CD8^+^ T cells. MHC-peptide dextramer^+^ cells were assessed within the gates representing CD4^+^ T cells and CD8^+^ T after substraction of corresponding negative control values. Initially, we correlated MHC-peptide dextramer^+^ cells at R0 and at any time-point of maximal response (Rmax) in HLA-A2^+^ patients (*n* = 12). Preexisting frequencies of CD8^+^ T cells specifically recognizing the E75 HER-2/neu peptide (CD8^+^/E75^+^ cells) were induced by the vaccine from a median of 0.59 % (range 0.15–1.49) at R0 to 1.61 % (range 0.18–2.98) at Rmax (*p* = 0.0025) (Fig. 3a). Frequencies of CD8^+^/E75^+^ cells at each time point (medians at R3: 0.50 %, R6: 0.83 % and LT: 1.72 %) are shown in Fig. 3b, with a statistical significant increase at time point LT vs R0 (*p* = 0.0177). Representative dot plots for negative control, R0 and Rmax for patient PR13 are depicted in Fig. 3c. The AE37 vaccine was also capable of increasing the frequencies of preexisting CD8^+^/PSA_146_^+^ cells from a median of 0.15 % (range 0.04–0.19) at R0 to 0.23 % (range 0.1–0.44) at Rmax (*p* = 0.0086) (Fig. 3d). CD8^+^/PSA_146_^+^ cells at each time point (medians at R3: 0.09 %, R6: 0.16 %, LT: 0.27 %) are shown in Fig. 3e, with a statistically significant increase at time point LT vs R0 (*p* = 0.0547). Representative dot plots for negative control, R0 and Rmax for patient PR6 are depicted in Fig. 3f. In both cases, percentages of dextramer^+^ CD4^+^ T cells, representing specificity controls, were minimal (Fig. 3b and e).

CD8^+^/HER2_85_^+^ T cell frequencies at R0, were close to the detection limit of 0.1 % in 8 out of 12 patients (range: 0.02–0.08 % of CD8^+^ T cells), whereas in 2 of them, levels were increased upon vaccination (R0 vs Rmax: 0.08 % vs 0.11 % and 0.06 % vs 0.14 %) (Additional file 1: Figure S1A). In the remainder 4 out of 12 patients, CD8^+^/HER2_85_^+^ cells at R0 were at relatively high numbers (0.10–0.19 % of CD8^+^ T cells) and in one of them, vaccination increased frequency levels from 0.17 to 0.40 %. For the total of 12 patients, CD8^+^/HER2_85_^+^cells were increased by the vaccine from a median 0.06 % (range: 0.02–0.19 %) at R0 to 0.12 % (range 0.04–0.4) at Rmax (*p* = 0.0025) (Additional file 1: Figure S1A). The levels of specific CD8^+^ T cells against HER2_435_, PSMA, SURV_96_ and TERT were at marginal levels at all time-points for the majority of patients, not allowing further evaluation (Additional file 1: Figure S1B-E).

**Fig. 4:**
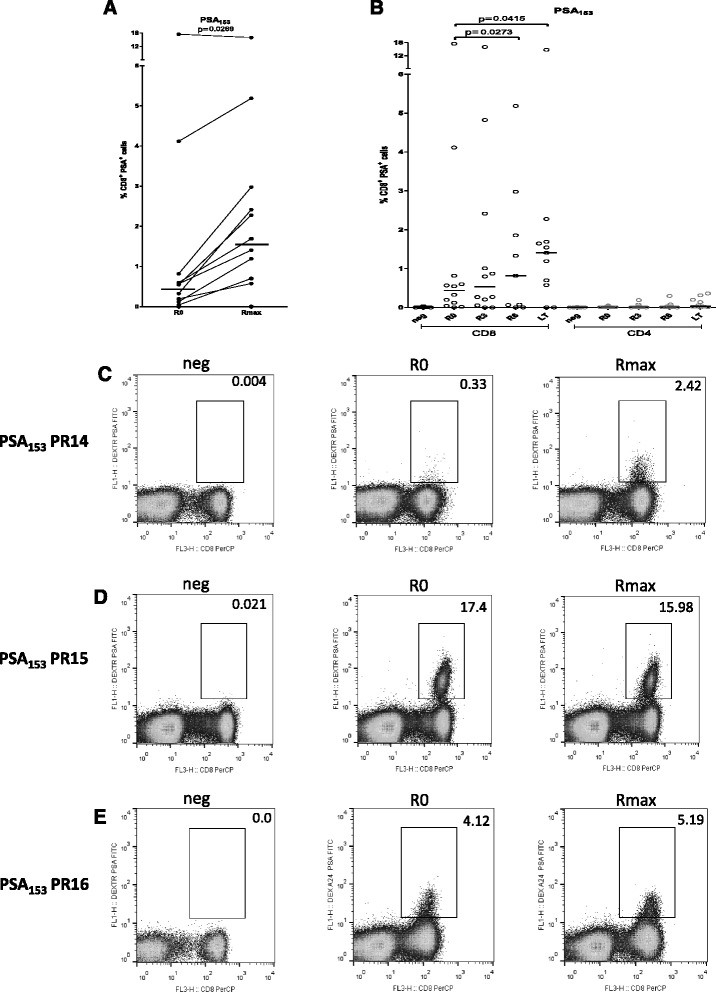
Preexisting and AE37-induced immunity to PSA_153_ in HLA-A24^+^ patients. Vaccinations induced, in a statistically significant manner, CD8^+^/PSA_153_^+^ cells (**a**). Frequencies of CD8^+^/PSA_153_^+^ cells at each time point (R0, R3, R6 and LT) (**b**). Representative dot plots for negative control, R0 and Rmax for patients PR14, PR15, PR16 (**c**-**e**)

Next, we correlated MHC-peptide dextramer^+^ cells at R0 and Rmax in HLA-A24^+^ patients (*n* = 12). CD8^+^/PSA_153_^+^ cells were induced by the vaccine from a median of 0.44 % (range 0.0–17.4) at R0 to 1.55 % (range 0.0–15.98) at Rmax (*p* = 0.0269) (Fig. 4a). Percentages of CD8^+^/PSA_153_^+^ T cells at each time point (medians at R3: 0.53 %, R6: 0.82 %, and R7: 1.41 %) are shown in Fig. 4b, with statistical significant increases compared to baseline at time points R6 and LT (*p* = 0.0273 and *p* = 0.0415, respectively). Representative dot plots for negative control, R0 and Rmax for patient PR14 are presented in Fig. 4c. Interestingly, prior to vaccination two patients exhibited high percentages of CD8^+^/PSA_153_^+^ cells that either remained unaltered or slightly increased (Fig. 4d, e). In the majority of patients, CD8^+^/SURV_20_^+^ T cells were detected at levels below 0.1 % prior to vaccination with a median of 0.06 % (range 0.01–0.28) (Additional file 1: Figure S1F).

Concluding, HLA-A2^+^ patients demonstrated preexisting immunity for E75 and PSA_146_ and HLA-A24^+^ patients for PSA_153_, which was enhanced upon vaccination with AE37 in a statistically significant manner.

### How preexisting immunity to HLA-A2 and HLA-A24-restricted CTL epitopes affects progression free survival

**Fig. 5:**
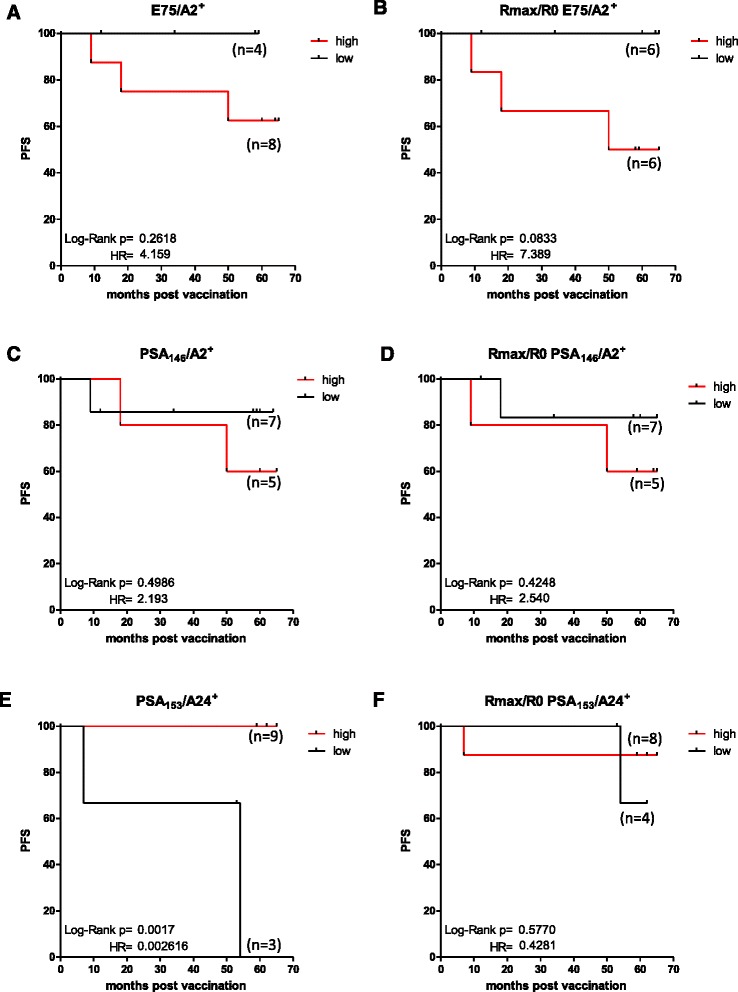
Preexisting immunity to HLA-A2 and HLA-A24-restricted CTL epitopes affects progression free survival. HLA-A2^+^ patients having high preexisting immunity to E75 showed a trend for shorter mPFS compared to them with low one (**a**). Patients with high Rmax/R0 had a trend for decreased mPFS, compared to patients with low Rmax/R0 (**b**). No statistically significant differences were observed among patients having high or low preexisting immunity to PSA_146_ and high or low Rmax/R0 (**c**, **d**). HLA-A24^+^ patients having high preexisting immunity to PSA_153_ showed statistically significant longer mPFS compared to patients with low (**e**). No statistically significant differences were observed among patients having high or low Rmax/R0 (**f**)

HLA-A2^+^ patients were grouped according to preexisting immunity to E75 and PSA_146_. More specifically, patients exhibiting levels of CD8^+^/E75^+^ T cells ≥0.41 % were considered as having high and those below 0.41 % as low preexisting immunity (x-tile software, Methods section). With a median follow up of 58 months for all patients, mPFS of patients with high (*n* = 8) or low (*n* = 4) preexisting immunity to E75 were not reached, although we observed a trend for shorter PFS among those who had high preexisting immunity (*p* = 0.2618, HR = 4.159) (Fig. 5a). Next, we correlated the ratio Rmax/R0 for % CD8^+^/E75^+^ cells with mPFS. Patients with high Rmax/R0 ratio (>2; *n* = 6) had a strong trend for decreased PFS, compared to patients with low Rmax/R0 ratio, albeit mPFS was also not reached in the latter group (*p* = 0.0833, HR = 7.389) (Fig. 5b). mPFS of patients having high (≥0.06 %) or low (<0.06 %) CD8^+^/PSA_146_^+^ cells (Fig. 5c) and high (>2) or low (≤2) ratio Rmax/R0 (Fig. 5d) were not reached, and no statistically significant differences were observed among groups.

HLA-A24^+^ patients were grouped according to preexisting immunity to PSA_153_. mPFS of patients having high (>0.04 %, *n* = 9) preexisting immunity to PSA_153_ was significantly higher (i.e. not reached) compared to patients with low (≤0.04 %, *n* = 3) (i.e. 54 months) (*p* = 0.0017, HR = 0.0026) (Fig. 5e). Finally, we correlated mPFS with the Rmax/R0 of CD8^+^/PSA_153_^+^ cells. There was no difference in mPFS of patients with high (*n* = 8) or low ratio (*n* = 4) (*p* = 0.5770, HR = 0.4281) (Fig. 5f).

Regarding the HLA-A2^+^ patients, preexisting immunity to E75 or PSA_146_, might correlate with shorter PFS. On the contrary, longer PFS seems to apply for HLA-A24^+^ patients with preexisting immunity to PSA_153._

## Discussion

Preexisting immunity along with epitope spreading are considered important factors securing an effective antitumor response, especially in the context of immunotherapeutic peptide vaccination. Beside this, others propose preexisting host immunity before vaccination as a basis for the design of efficient vaccination protocols [14, 15]. In line with this, we have previously shown that AE37 vaccination offered a clinical benefit in prostate cancer patients, with high levels of preexisting immunity to the native AE36 peptide detected by IFNγ ELISPOT [13]. It is worth mentioning, that AE37 is a multipepitope vaccine, capable of inducing both specific CD4^+^ and CD8^+^ T cells in vaccinated cancer patients [9]. The AE37 vaccine encompasses the immunogenic epitope of HER-2 p776-790 (the 15-mer AE36) which is subjected to the Ii-key modification for enhancing recognition by CD4^+^ T cells and also shows highly promiscuous binding to a series of MHC class II alleles with various affinities, as tested in binding and functional assays [44–46]. HLA class II-matched peptides such as AE36, may be particularly suited for activating CD4^+^ T cells in vaccination protocols thereby enabling their extensive interactions with other immune cells [6, 47]. We hypothesize that AE37-induced T-helper cells may engage dendritic cells at tumor sites, thereby cross-presenting antigens from apoptotic tumor cells and inducing epitope spreading.

Herein, we have addressed this issue by testing on our long-term survivors from the phase I trial for vaccine-induced epitope spreading. To this end, we have used MHC class I dextramers to identify both at baseline (i.e. preexisting) and during vaccinations, T cell responses (measured as % of dextramer-specific CD8^+^ T cells) against other HER-2/neu epitopes or against epitopes from other tumor antigens, representing intramolecular and intermolecular spreading, respectively. Because epitope spreading reflects an endogenous immunologic response closely related to the broader spectrum of tumor-specific preexisting immunity, we also analyzed our vaccinated patients for preexisting immunity to the vaccine by AE36-specific IFNγ-based ELISPOT assay and by LR1. We also planned to evaluate whether preexisting immunity to AE36 in combination with epitope spreading was predictive of treatment benefit. With respect to the latter, we analyzed frequencies of CD8^+^ T cells recognizing CTL specific epitopes restricted by HLA-A2 or HLA-A24, which are the most commonly expressed alleles among our study patients. It has to be mentioned that statistical significance could not be reached in many instances mostly due to the very limited patient numbers compared in each subgroup. However consistent trends can be interpreted as proof-of-principle data and require further consideration. Our data demonstrated that patients with high preexisting immunity to AE36, irrespectively of HLA-A2 or A24 expression, showed statistically significant longer PFS, than patients with low AE36 preexisting immunity, in accordance with our previous observation of improved OS in these patients with high baseline IFN-γ response to the peptide AE36 [10]. Similar results were also obtained in a phase II clinical trial of breast cancer patients vaccinated with AE37 in the adjuvant setting [48]. To our knowledge, this is the first observation which renders preexisting immunity to a long peptide vaccine as a predictive biomarker, for the selection of patients most likely to benefit clinically from vaccination.

Local reactions at different vaccinations cycles have been recently correlated with improved clinical outcome [30, 31]. Here, we propose for the first time LR1, i.e. the local reaction (induration) 48 h after the first vaccine, indicative of preexisting antitumor immunity, as a predictive surrogate biomarker for immunological and clinical responses in patients undergoing injections with AE37 and immunoadjuvant.

Based on our data, we detected preexisting immunity for several HLA-A2 and HLA-A24 restricted tumor epitopes in patients expressing the respective alleles, which was actually enhanced post AE37 vaccination. Of the epitopes analyzed, the HLA-A2 restricted E75 and PSA_146–151_ peptides as well as the HLA-A24 restricted PSA_153–161_ peptide were the most immunogenic ones based on their relatively high frequencies of CD8^+^ T cells (>0.15 %) at baseline, which were even further augmented during vaccinations. To unravel possible predictive significance of preexisting immunity for clinical outcome in our vaccinated patients, we correlated frequencies of CD8^+^ T cells, at baseline and during vaccinations, specific for the E75, PSA_146–151_ or PSA_153–161 _peptides, with mPFS of patients carrying the appropriate HLA restricting alleles. Our analyses showed that high levels of preexisting immunity to PSA_153–161_ correlated with significantly higher mPFS in HLA-A24^+^ patients, whereas no such correlation was observed in HLA-A2 carriers with preexisting immunity to E75 or PSA_146–154_ peptides. In contrast to our expectations, HLA-A2^+^ patients with high preexisting or vaccine-induced immunity to E75, showed a trend for shorter PFS than patients with low levels of such immunity. This observation could be explained by the assumption that although the immune system of these patients had a preexisting memory for E75 and responded by epitope spreading post vaccination, increasing epitope specific CTLs, they failed to interpret this to an effective clinical response, due to possible changes of the tumor immune profile. In line with this, results from a clinical vaccination study in melanoma patients, showed that no significant correlation was observed between clinical response and increases in the post vaccination peptide specific CD8^+^ tetramer^+^ T cells [49]. Despite the fact that HLA-A2 may be an adverse prognostic factor in prostate cancer [50], it has been previously described that HLA-A2 along with HLA-C3 can predict prevention of relapse in melanoma patients vaccinated with Melacine [51]. Moreover, encouraging results have been reported in a previous study, where HLA-A2 breast cancer patients received trastuzumab therapy concomitantly with a HER2/neu T-helper peptide-based vaccine, encompassing HLA-A2 immunogenic motifs, including E75 [25].

It is well known that altered expression of HLA class I, ranging from total loss to reduced expression of single loci and alleles, has been found in high frequency in several cancer types [52], including also prostate cancer [53], and is a mechanism which accounts for the selective outgrowth of tumor-escape variants during immunoediting [54–58]. Moreover, patients with prostate cancer and HLA class I abnormalities in their lesions, have poorer clinical outcome, than those with no detectable HLA class I antigen abnormalities in their tumors [59].

HLA-A*02 genotype has been reported to be a strong prognostic factor linked to the aggressiveness of ovarian cancer of serous histology, prostate cancer and malignant melanoma [60] with a selective loss found in these and other types of cancer [47–49]. The underlying mechanism for HLA-A2 loss has not been clarified yet. However, different hypotheses have been reported, such as up regulation of miR-181a, in different types of cancer [61–64], which has been previously associated with selective downregulation of HLA-A2 [65]. Another hypothetical explanation of the poor prognosis that characterizes HLA-A2^+^ patients, could be rather genetic than immunological [66].

Another possibility could be the well known consequences of HER-2 expression on MHC class I restricted antigen presentation machinery, which connect HER-2 overexpression with downregulation of surface MHC class I expression [67–70]. These defects in components of the antigen processing and presentation machinery, induced by HER-2, obstruct the in vivo generation of class I restricted HER-2 derived epitopes, haltering tumor recognition by CTL [71]. To this end, Norell et al. showed that metastatic ovarian tumor cells altered HLA class I expression through haplotype loss which was associated with inefficient HLA-A2-restricted immunity to HER-2 [72].

A third option could be that tumors with high expression of HER-2, as it is the case with progressed castrate resistant prostate cancer [3–5], are often characterized by alleviated capacity of being recognized by tumor antigen-specific CTL. It has been previously reported that especially for E75 immunization protocols, E75 specific vaccine-induced CTLs failed to recognize HER-2^+^ tumors [73] and HLA-A2^+^-HER-2 overexpressing carcinomas, even after IFNγ treatment [74]. This could explain our observation that patients with high preexisting immunity against E75 and patients with induced E75-immunity upon vaccination with AE37 demonstrated shorter PFS.

Our data are also supporting the notion that preexisting AE36 immunity might be beneficial through induction of antigen specific CD4^+^ T cells, with cytotoxic function, that could successfully recognize tumor cells with down-regulated HLA class I alleles. Vaccine- induced CD4^+^ T cells with cytotoxic antitumor activities have been previously described [12, 75, 76]. Another important aspect of preexisting AE36 immunity is the local production of cytokines, such as IFNγ, by activated CD4^+^/AE36^+^ cells, which could not only restore tumor cells’ HLA class I loss, but also activate components of innate immune system against tumor cells (e.g. NK cells, eosinophils, macrophages and neutrophils).

## Conclusions

Our data although hypothesis generating, still they raise a very important issue by introducing an ambiguity whether preexisting immunity or epitope spreading specific for HLA-class I-restricted peptides can actually predict a favorable clinical outcome. They further strengthen the notion that peptide vaccines should contain HLA class II epitopes aiming at the activation of Th cells in order to counteract selective outgrowth of HLA class I negative tumor variants, under the pressure of immune selection mediated by tumor specific cytotoxic T cells. Thus, preexisting immunity to long vaccine peptides encompassing HLA class II, but also HLA class I epitopes, may offer an advantage in the clinical outcome of vaccinated patients, further contributing to the improvement of future immunotherapeutic protocols.

## Additional file

Additional file 1: Figure S1. Frequencies of CD8^+^ cells recognizing HER_85_ (A), HER_435_ (B), PSMA (C), SURV_96_ (D), TERT (E) in HLA-A2^+^ patients and SURV_20_ (F) in HLA-A24^+^ patients, at time points R0 and Rmax. Statistical significant increase was observed for HER_85_ and TERT, while a strong trend was observed for SURV_96_. (PPTX 249 kb)

## Abbreviations

APC: Antigen presenting cells
CTL: Cytotoxic T lymphocytes
DTH: Delayed-type hypersensitivity
GM-CSF: Granulocyte–macrophage colony-stimulating factor
HLA: Human Leukocyte Antigen
IFNγ: Interferon gamma
LR1: Local reaction after 1st vaccination
LT: Long term
MHC: Major histocompatibility complex
NK: Natural killer cells
OS: Overall survival
PBMC: Peripheral blood mononuclear cells
PSA: Prostate specific antigen
PSMA: Prostate specific membrane antigen
TERT: Telomerase reverse transcriptase
Th: T helper cells
