# Supplementary figures and images for: Unraveling the role of preexisting immunity in prostate cancer patients vaccinated with a HER-2/neu hybrid peptide
Source: J Immunother Cancer. 2016 Nov 15;4:75. doi: 10.1186/s40425-016-0183-4 (PMC5109671; doi:10.1186/s40425-016-0183-4)

## Slide 1
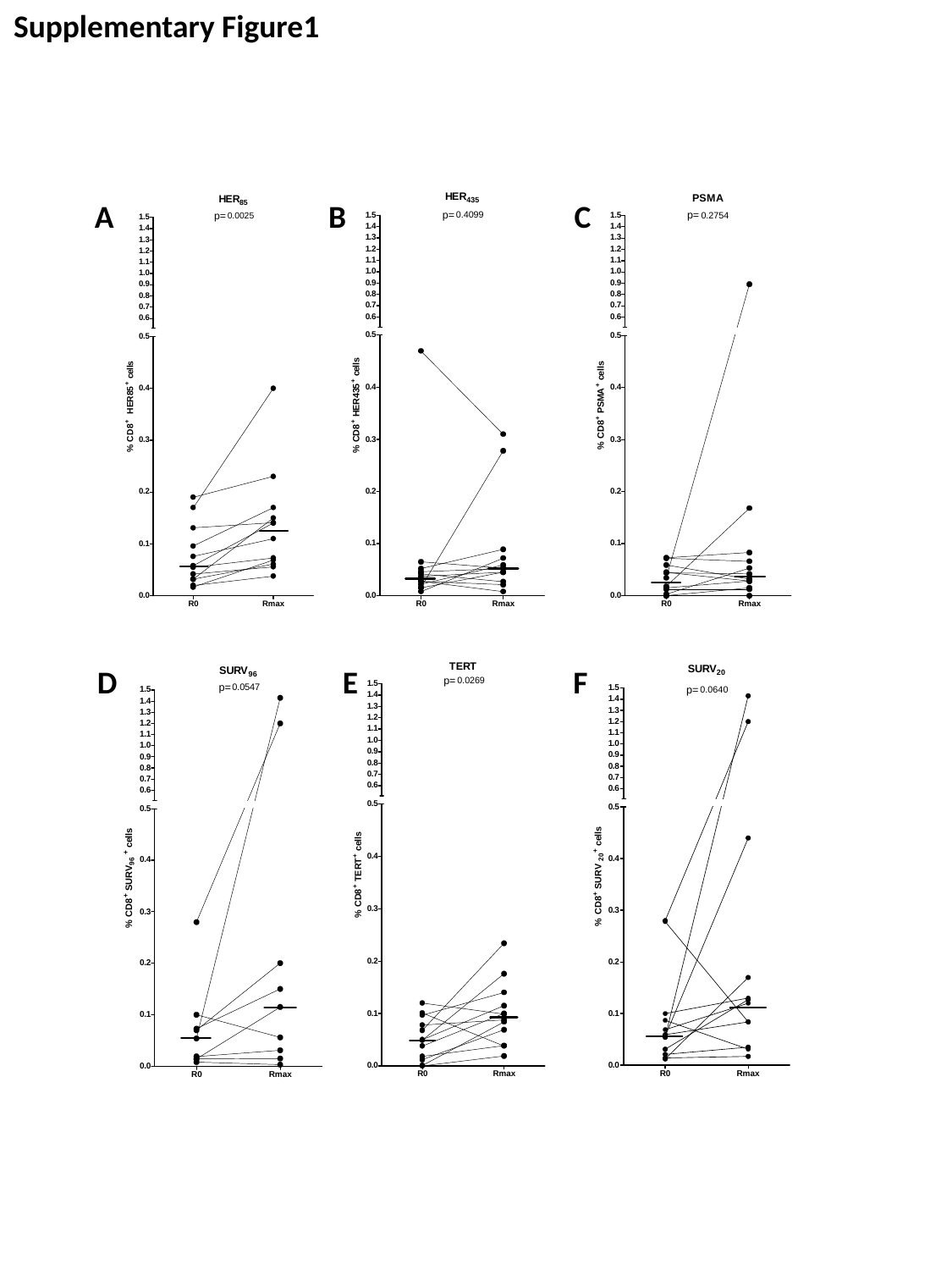

Supplementary Figure1
A
B
C
D
E
F

Supplement: Additional file 1: Figure S1. — Frequencies of CD8+ cells recognizing HER85 (A), HER435 (B), PSMA (C), SURV96 (D), TERT (E) in HLA-A2+ patients and SURV20 (F) in HLA-A24+ patients, at time points R0 and Rmax. Statistical significant increase was observed for HER85 and TERT, while a strong trend was observed for SURV96. (PPTX 249 kb) [file 40425_2016_183_MOESM1_ESM.pptx]
